# Quality Assessment of MRI-Radiomics-Based Machine Learning Methods in Classification of Brain Tumors: Systematic Review

**DOI:** 10.3390/diagnostics14232741

**Published:** 2024-12-05

**Authors:** Shailesh S. Nayak, Saikiran Pendem, Girish R. Menon, Niranjana Sampathila, Prakashini Koteshwar

**Affiliations:** 1 Manipal College of Health Professions, Manipal Academy of Higher Education, Manipal 576104, Karnataka, India; shailesh.nayak@manipal.edu (S.S.N.); saikiran.p@manipal.edu (S.P.); 2 Kasturba Medical College, Manipal Academy of Higher Education, Manipal 576104, Karnataka, India; girish.menon@manipal.edu; 3 Manipal Institute of Technology, Manipal Academy of Higher Education, Manipal 576104, Karnataka, India; niranjana.s@manipal.edu

**Keywords:** Glioma, differential diagnosis, radiomics, radiomics quality score, texture analysis

## Abstract

Background: Brain tumors present a complex challenge in clinical oncology, where precise diagnosis and classification are pivotal for effective treatment planning. Radiomics, a burgeoning field in neuro-oncology, involves extracting and analyzing numerous quantitative features from medical images. This approach captures subtle spatial and textural information imperceptible to the human eye. However, implementation in clinical practice is still distant, and concerns have been raised regarding the methodological quality of radiomic studies. Methodology: A systematic literature search was performed to identify original articles focused on the use of radiomics for brain tumors from 2015 based on the inclusion and exclusion criteria. The radiomic features train machine learning models for glioma classification, and data are split into training and testing subsets to validate the model accuracy, reliability, and generalizability. The present study systematically reviews the status of radiomic studies concerning brain tumors, also using the radiomics quality score (RQS) to assess the quality of the methodology used in each study. Results: A systematic search of PubMed identified 300 articles, with 18 studies meeting the inclusion criteria for qualitative synthesis. These studies collectively demonstrate the potential of radiomics-based machine learning models in accurately distinguishing between glioma subtypes and grades. Various imaging modalities, including MRI, PET/CT, and advanced techniques like ASL and DTI, were utilized to extract radiomic features for analysis. Machine learning algorithms such as deep learning networks, support vector machines, random forests, and logistic regression were applied to develop predictive models. Conclusions: The present study indicates high accuracies in glioma classification, outperforming traditional imaging methods and inexperienced radiologists in some cases. Further validation and standardization efforts are warranted to facilitate the clinical integration of radiomics into routine practice, ultimately enhancing glioma management and patient outcomes. Open science practices: Machine learning using MRI radiomic features provides a simple, noninvasive, and cost-effective method for glioma classification, enhancing transparency, reproducibility, and collaboration within the scientific community.

## 1. Introduction

In the context of medical publications, it is crucial for general medical practitioners to possess a foundational understanding of the diagnosis and management of brain tumors due to their prevalence. The most common forms of brain tumors include intracranial metastases from systemic cancers, meningiomas, and gliomas, notably glioblastoma [1]. The development of this diverse group of tumors lacks specific etiological or risk factors. The molecular characterization of brain tumors indeed offers new insights, but it also highlights the limitations of previous studies, which typically relied on histopathology and imaging for tumor classification and prognosis. Previous studies primarily categorized brain tumors based on morphological features visible under the microscope. However, this approach often overlooked molecular heterogeneity, leading to a lack of precise subclassification. Molecular profiling now reveals that tumors with similar histological features may have distinct genetic profiles, which can influence their behavior and treatment response. Nevertheless, the current focus on the molecular characterization of brain tumors offers fresh insights into the genetic aberrations that form the basis of their tumorigenesis [2].

The most prevalent primary brain tumors, gliomas, originate from the glial cells, encompassing astrocytes, oligodendrocytes, and ependymal cells [3]. In accordance with the World Health Organization (WHO) classification [4], gliomas are divided into two distinct categories grounded in their malignant potential: low-grade glioma (LGG) for grades I to II, presenting with focal symptoms, and high-grade glioma (HGG) for grades III to IV, manifesting with generalized symptoms. Notably, glioblastoma (GBM), designated as a grade IV tumor, accounts for 54% of the total glioma cases, underscoring its significance in the spectrum of gliomas [5].

Brain imaging plays a crucial part in glioma management, facilitating precise diagnosis, cataloguing, surgical planning, and post-treatment follow-up. Typically, the initial imaging modality for diagnosing glioma is a brain computed tomography (CT) scan, revealing a hypodense lesion, often exhibiting rim enhancement with contrast agent injection. While CT offers valuable anatomical information, it is commonly tailed by magnetic resonance imaging (MRI), which is often regarded as having better contrast resolution. MRI can provide complementary information, enhancing the overall diagnostic capability in glioma cases [6,7].

Current research has demonstrated that traditional MRI sequences are still viable, particularly gadolinium-based contrast-enhanced T1-weighted imaging (T1-CE), in the grading of gliomas [8]. Technological advancements have further introduced advanced MRI sequences that play a role in physiological and metabolic assessments for glioma classification, including perfusion-weighted imaging (PWI) and diffusion-weighted imaging (DWI) [9,10]. It is worth noting that prior studies on glioma grading faced limitations, as they relied on a small number of parameters extracted from a single MRI sequence [8,9,10].

Radiomics is the process of the computer-assisted extraction of quantitative information from radiological images, typically in the form of sub-visual radiographical signals [11,12]. This approach allows the creation of mineable databases that can be employed for various purposes, including diagnosis, the characterization of prognosis, and the evaluation or prediction of therapeutic responses [8].

The radiomic concept hinges on the idea that biomedical imaging obtained from medical modalities like CT, MRI, and PET harbors concealed information, discoverable through quantitative image analyses. This information serves to complement the data interpreted by radiologists and provides additional pathophysiological insights [13,14,15]. Leveraging advanced mathematical algorithms, radiomics excels in uncovering a broader spectrum of tumor features that may elude visual detection. At its core, radiomics operates on the fundamental principle that pathological processes altering tissue characteristics result in modifications in pixel intensity and distribution. These alterations are reflected in unique values of textural features compared to those observed in normal tissue or tissues affected by other diseases [9,10].

Radiomics, which extracts quantitative features from medical images, holds great potential in glioma classification and prognosis prediction. However, there are several limitations to its use, especially when combined with machine learning methods. One major challenge is the inconsistency in image acquisition and preprocessing, which can affect the reproducibility and generalizability of the extracted features. Differences in imaging protocols, such as variations in scanning equipment or settings, can lead to discrepancies that hinder the model’s performance across diverse datasets or clinical environments. Additionally, the high dimensionality of the data can lead to overfitting in machine learning models if feature selection and dimensionality reduction are not carefully managed [16].

Over the past decade, there has been a substantial increase in radiomic studies dedicated to displaying the effectiveness of radiomic features in glioma classification and distinguishing gliomas from metastases, PCNLS, and non-neoplastic brain diseases. Despite extensive studies linking quantitative image features to diagnosis, response evaluation, and prognostic prediction in glioma patients using CT and MRI, at present, the application of radiomics in distinguishing gliomas is primarily found in scholarly publications. Clinicians are skeptical about the validity of radiomics in this context, largely because it has not been effectively translated into therapeutic applications. Assessing the clinical value of radiomics in gliomas has proven challenging due to the complexity of methods and varying study designs. Hence, we conducted this study to evaluate the methodological quality of the existing research, utilizing the “radiomics quality score” (RQS).

## 2. Materials and Methods

MRI of the brain is predominantly used for the evaluation of gliomas. Radiomics has been an active research area in medical image processing and analysis. Currently, MRI-based radiomic features and machine and deep learning classification models are employed for glioma grading. Radiomic-feature-based machine learning models for glioma classification involve extracting quantitative imaging features from MRI brain images to classify them into different subtypes, typically based on their malignancy or genetic characteristics.

The integration of MRI radiomic features and machine learning can revolutionize glioma classification, offering a noninvasive, reproducible, and potentially personalized approach to diagnosis, prognosis, and treatment planning. However, further studies, model validation, and clinical trials are necessary to ensure safe and effective implementation in routine clinical practice. In the current study, we analyzed a dataset of several glioma patient studies that included distinct molecular subtypes and was gathered from multiple clinical centers. Based on their accuracy, we show that several ML models can categorize gliomas into their molecular subtypes, such as astrocytoma, oligodendroglioma, and glioblastoma, with a high degree of sensitivity and specificity.

A systematic search was meticulously conducted to identify all published studies utilizing radiomics for glioma, employing the most pertinent scientific electronic databases, notably PubMed. The search strategy was meticulously crafted and applied comprehensively. Only studies published since 2015 were considered, with the last search executed on 1 November 2023. The search parameters incorporated key terms outlined in the Supplementary Materials. The literature search was constrained to English-language publications, subjects aged 18 years and above, studies involving human subjects, and studies with full-text availability.

After two reviewers independently screened recognized titles and abstracts, they carefully examined the entire texts of papers that addressed the application of radiomics in glioma grading in relation to other disorders, leaving out review articles. More selection criteria were used for publications that fit these parameters and could be accessed in full.

### 2.1. Inclusion and Exclusion Criteria

The following inclusion criteria were used to choose studies: (a) original research articles with patients whose diseases were confirmed by pathology and surgery or thorough analyses of medical history, clinical symptoms, and different imaging data along with details about the imaging protocol; (b) patients with WHO-grade gliomas that were histopathologically confirmed, including both low-grade and high-grade gliomas; and (c) the utilization of machine learning (ML) with radiomic features applied for classifying gliomas.

Exclusion criteria were applied for studies that (a) did not employ ML for glioma grade classification; (b) did not specifically focus on differentiating between lower-grade gliomas (LGGs) and high-grade gliomas (HGGs); and (c) had a small sample size that hindered the application of machine learning classifiers.

**Figure 1:**
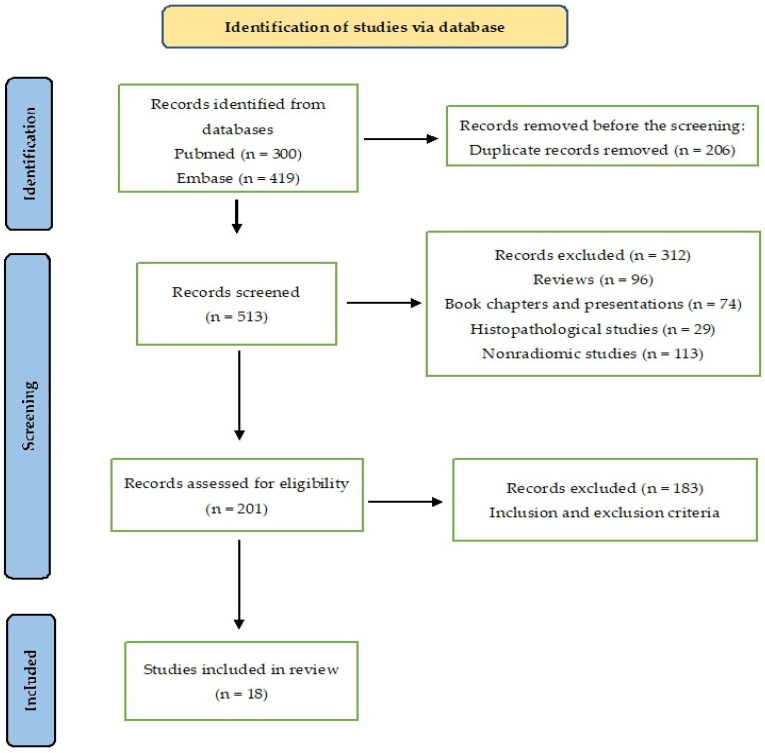
The PRISMA flow diagram for study selection.

This systematic review adhered to the Preferred Reporting Items for Systematic Reviews and Meta-Analyses (PRISMA) statement guidelines (Figure 1).

### 2.2. Data Extraction

The authors S.N.S. and S.K.P. conducted a thorough data extraction process for the systematic review, gathering essential information such as author names, publication years, study locations, target populations, study designs and sizes, machine learning techniques employed, modalities utilized for feature extraction, and interpretations of study results. This meticulous procedure ensured comprehensive coverage and accuracy in synthesizing the research findings.

### 2.3. Quality Assessment

Two reviewers, P.K. and N.S., evaluated each included study using the radiomics quality score (RQS), a tool comprising 16 items designed to assess the key aspects of the radiomics analysis workflow (Supplementary Materials). Discrepancies between reviewers were resolved through consensus. Each study was evaluated based on the quality of image protocols, the segmentation techniques employed, and the rigor of validation strategies, following the RQS guidelines. The validity of the RQS was assessed by sharing it with two experts, each with over 10 years of experience in MRI, image processing, and segmentation. Their feedback and comments were incorporated to create the final draft for evaluating the overall RQS of the articles. The RQS evaluates three main checkpoints: the first checkpoint assesses “image protocol quality”, with a maximum score of 2 points; the second checkpoint, consisting of three items related to segmentation strategies, phantom use, and imaging time points, allows for up to 3 points; and the third checkpoint, comprising 12 items covering feature extraction, exploratory analysis design, and model building/validation, offers a maximum of 31 points. The total score ranges from −8 to 36, which can be converted into a final RQS percentage ranging from 0 to 100. For detailed RQS checkpoints, items, and corresponding points, please refer to Supplementary Tables S1 and S2.

### 2.4. Data Synthesis and Analysis

The radiomic features extracted from the tumor volume using various segmentation tools were subjected to dimensionality reduction to identify the most effective features for distinguishing between gliomas. These selected features were then used to train and validate the dataset through machine learning and deep learning methods. Please refer to Supplementary Tables S3 and S4.

We meticulously tabulated the outcomes of each study to facilitate the identification of emerging patterns within the data. Given the substantial diversity in study design, reported outcomes, and outcome metrics, we opted for a narrative data synthesis rather than a formal quantitative meta-analysis. This approach allowed for a comprehensive analysis of the data while acknowledging their inherent heterogeneity.

## 3. Results

### 3.1. Literature Search

A total of 300 articles were obtained by searching a scientific electronic database (PubMed) using the key terms (Supplementary Materials). A comprehensive assessment was conducted on a total of 118 articles. Among these, 42 studies were subjected to initial screening and further underwent a second round of screening. After a meticulous examination of the complete content, a refined selection process culminated in the inclusion of 18 records deemed suitable for qualitative synthesis. The PRISMA flow diagram of included studies according to the inclusion and exclusion criteria is presented in Figure 1.

### 3.2. Characteristics of Included Studies

The included studies (Table 1) present a diverse array of characteristics in terms of the study design, sample size, imaging modalities, and machine learning algorithms employed. They collectively form a rich body of evidence highlighting the potential of radiomics in glioma grading and differential diagnosis.

Among the studies, there is a mixture of single-center and multicenter investigations, with sample sizes ranging from relatively small cohorts of around fifty patients to larger studies involving hundreds or even thousands of participants. This diversity allows for a broad exploration of radiomics methodologies across different settings, enhancing the generalizability of findings. The imaging modalities utilized include various sequences of MRI such as T1-weighted, T2-weighted, T1 contrast-enhanced, FLAIR, and diffusion-weighted imaging (DWI). Additionally, some studies incorporate advanced imaging techniques like PET/CT and ASL for the comprehensive characterization of gliomas. This multi-modality approach enables a more comprehensive analysis of tumor features, enhancing diagnostic accuracy and prognostic value. The machine learning algorithms employed encompass a wide range of techniques, including deep learning (DL) networks, support vector machines (SVMs), random forests (RFs), logistic regression (LR), and gradient boosting classifiers (GBCs), among others. These diverse methodologies reflect the complexity of glioma classification and the need for robust predictive models to handle the intricacies of radiomic data.

Overall, these studies collectively underscore the potential of radiomics as a noninvasive, quantitative tool for glioma characterization, with the promise of improving diagnostic accuracy and prognostic stratification and guiding personalized treatment decisions. However, further validation and standardization efforts are warranted to ensure the clinical utility and widespread adoption of radiomics in routine practice.

### 3.3. Radiomics for Glioma Grading and Differential Diagnosis

Based on the studies provided (Table 2), radiomics has emerged as a powerful tool for glioma grading and differential diagnosis. These studies utilized various imaging modalities, including MRI, PET/CT, and advanced techniques like ASL and DTI, to extract radiomic features for analysis. Machine learning algorithms such as deep learning networks, support vector machines, random forests, and logistic regression were applied to these radiomic datasets to develop predictive models for glioma classification.

Luo et al., 2020 [11], conducted a multicenter study involving 655 glioma patients and employed a DL network (3D U-net) and IS-based method to stratify IDH-wild-type lower-grade glioma and triple-negative glioblastoma, achieving high accuracies in both cross-validation and independent testing cohorts. Similarly, Nakamoto et al., 2019 [12], demonstrated the effectiveness of LR, SVM, SNN, RF, and NB models in preoperatively grading malignant gliomas using CE-T1WIs and T2WIs.

Gutta et al., 2021 [13], focused on gliomas and employed SVM, RF, and gradient boosting algorithms trained with radiomic features, with the CNN achieving an average accuracy of 87%, and Giorgio Russo et al., 2021, investigated the role of neural networks, RF, SVM, and generalized linear models in predicting the grading of primary brain tumors using 11[C]-MET PET/CT scans. Additionally, Zhang et al., 2020 [14], utilized an SVM classifier with a linear kernel to distinguish LGGs from HGGs with high accuracy based on radiomic features extracted from DTI images.

Furthermore, Zhu et al., 2023 [15], and Guo et al., 2023 [17], investigated the role of MLP, SVM, RF, and logistic regression in discriminating between HGGs and LGGs, with MLP outperforming 3D-ASL and DWI improving the performance of conventional MRI-based radiomic models for classifying gliomas based on their molecular subtypes.

In addition to these studies, Zhang et al., 2021 [18], showed the utility of an MRI-radiomics-based random forest model in differentiating GBM from LGG, outperforming inexperienced radiologists. Moreover, Hashido et al., 2021 [19], highlighted the usefulness of radiomics-based machine learning classifiers using quantitative ADC and CBF maps in differentiating HGGs from LGGs.

In 2023, Kumar et al. [20] examined the performance of random forest (RF), support vector machine (SVM), gradient boosting classifier (GBC), Naive Bayes Classifier (NBC), and Ada-Boost Classifier (ABC) in differentiating low-grade gliomas from high-grade gliomas using T2-weighted MRI sequences. Similarly, He et al. conducted a multicenter study in 2022 involving various machine learning models (SVM, autoencoder, RF, LDA, LR, LR-Lasso, DT) and MRI sequences (T1, T2, FLAIR, T1Gd) to predict the molecular subtypes of gliomas. Furthermore, J et al. [21] in 2022 utilized multiparametric DWI models to differentiate low-grade gliomas from high-grade gliomas with better generalization performance than single DWI models.

Ding et al. [22] utilized radiomic features extracted from multiplanar reconstructed (MPR) images and deep learning features to construct classification models for glioma grading. Their study emphasized the importance of utilizing multiplanar imaging features and combining radiomics with deep learning for accurate glioma classification.

Bonte et al. [23] leveraged radiomic features extracted from [18F] FET PET and MRI scans to discriminate between LGGs and HGGs, achieving high accuracy by combining features from both modalities. While Ning et al. [24] integrated radiomics and deep features from MRI scans to develop a grading model, achieving high diagnostic performance in differentiating between high-grade and low-grade tumors, Park et al. [25] focused solely on analyzing radiomic features extracted from MRI scans to predict glioma grades.

**Table 1:** Summary of various MRI-radiomics-based ML algorithms for glioma grading and differential diagnosis.

Authors and Year	Modalities Used for Feature Extraction	Classification Model
Luo et al., 2020 [11]	T1 contrast and T2 FLAIR series of MRI	DL network (3D U-net) and IS
Takahiro Nakamoto et al., 2019 [12]	T1-weighted MRI (CE-T1WI) and T2-weighted MRI (T2WI)	LR, SVM43, SNN44, RF45, and NB4
Gutta et al., 2021 [13]	T1-weighted, T1CE, and T2-weighted/FLAIR	SVM, RF, and gradient boosting trained with radiomic feature
Giorgio Russo et al., 2021 [26]	11[C]-MET PET/CT	Neural networks, RF, SVM, and generalized linear models
Zhang et al., 2020 [14]	T1 inversion recovery sequence, T2-weighted sequence, FLAIR sequence, and axial T1 contrast-enhanced sequence	SVM classifier with a linear kernel
Zhu et al., 2023 [15]	MRI T1WI contrast-enhanced images	MLP, SVM, RF, and logistic regression
Kumar et al., 2023 [20]	T2-weighted MRI sequences	RF classifier RFC, SVM, GBC, NBC, and ABC
He et al., 2022 [27]	MRI (T1, T2, FLAIR, and T1Gd)	SVM, AE, RF, LDA, LR, LR via lasso (LR-Lasso), and DT
J. et al., 2022 [21]	T1WI+C, T2W-FLAIR, and DWI sequences	Monoexponential, IVIM, DKI, FROC, CTRW, and stretched exponential using MATLAB
Guo et al., 2021 [28]	T2- and T1-weighted imaging, T2-weighted attenuated inversion recovery imaging (T2 FLAIR), and contrast-enhanced T1-weighted imaging	LASSO
Zhang et al., 2021 [18]	T1-weighted, T2-weighted, contrast-enhanced T1-weighted, and FLAIR	RF classification algorithm
Hashido et al., 2021 [19]	T1-weighted fluid-attenuated inversion recovery (T1-FLAIR), T2-FLAIR, T2WI, T2*WI, DWI, pCASL imaging, and contrast-enhanced T1WI	LASSO-LR, RF, SVM with radial basis function kernel (SVM-RBF), and SVM with linear kernel (SVM-L)
Ding et al., 2022 [22]	Contrast-enhanced T1-weighted imaging	SVM, LR, and RF
Ning et al., 2021 [24]	T1-weighted and T2 FLAIR MRI	A kernel-fusion-based SVM classifier was used to integrate these multi-modal features for grading gliomas
Park et al., 2019 [25]	T2-weighted images, T1C, and FLAIR	Elastic net, RF, GBM, and LDA algorithms
Bonte et al., 2018 [23]	T1CE and FLAIR MRI	RF

Abbreviations: ABC: Ada-Boost Classifier; AE: autoencoder; CTRW: continuous-time random walk; DT: decision tree; DKI: diffusion kurtosis imaging; GBC: gradient boosting classifier; IVIM: intravoxel incoherent motion; FLAIR: fluid-attenuated inversion recovery; LDA: linear discriminant analysis; LR: logistic regression; FROC: fractional order calculus; MLP: multilayer perceptron; NBC: Naive Bayes Classifier; IS: image signature; RF: random forest; SVM: support vector machine.

**Table 2:** Performance of MRI-radiomics-based ML algorithms for glioma grading and diagnosis and the sample size used.

No. of Patients	Task	Accuracy	AUC	Outcomes
655 glioma patients	Image-based (IS) method was employed to stratify IDH-wild-type lower-grade glioma and triple-negative glioblastoma	86.1 and 89.8 for validation cohorts; 83.9 and 80.4 for testing cohorts	-	Radiomic model demonstrates effectiveness in noninvasive histo-molecular pathological diagnosis and prognostic stratification of gliomas. This holds promise for its potential integration into routine clinical applications in the future.
224 malignant gliomas	Prediction models in the independent validation set	-	0.902 ± 0.024 0.747 ± 0.034	The presented framework demonstrates efficacy as a valuable tool for preoperative grading of malignant gliomas.
237 gliomas	CNN; top-performing ML model gradient boosting	87 64	-	CNN can learn discriminating features automatically and can provide substantial improvement in glioma grade prediction.
56 primary brain tumors	Overall accuracy in the entire patient dataset, comprising both Siemens and GE scans	64.13	70.31	The ML model was shown to be workable and capable of identifying radiomics aspects of the 11[C]-MET PET that might be useful in predicting the disease’s grade.
136 gliomas	Classification of LGGs and HGGs; classification of grade III and IV	0.94 0.98	0.93 0.99	Radiomic features extracted from the fractional anisotropy (FA) and mean diffusivity (MD) maps of brain diffusion tensor imaging (DTI) images offer significant utility in noninvasive grading, distinguishing between lower-grade gliomas (LGGs) and higher-grade gliomas (HGGs), as well as effectively classifying grade III and grade IV tumors.
105 gliomas	Verification group, MLP and maximum rCBFmax; test group, MLP and rCBFmax	-	0.968 and 0.815; 0.981 and 0.815	ML-based MLP classifier model exhibited superior performance in discriminating between HGGs and LGGs to 3D-ASL.
83 gliomas	RF model demonstrated superior performance	0.83	0.81	A model designed for the classification of gliomas into low-grade and high-grade categories.
108 gliomas				Promising prediction of the molecular subtypes; this study also provided a general tool for radiomics investigation.
74 gliomas	DWI model/single DWI model	-	0.84/0.71	Multiparametric DWI model exhibited superior generalization performance in distinguishing between LGG and HGG compared to the established single DWI model.
152 gliomas				The combined model and the radiomics signature both performed better than the clinical model and demonstrated good diagnostic effectiveness.
142 gliomas	Random forest analysis		1.00	The MRI-radiomics-based random forest model demonstrated utility in effectively distinguishing between glioblastoma (GBM) and lower-grade glioma (LGG).
52 gliomas	LASSO-LR, RF, and SVM-RBF models for training and testing	-	0.965, 1.000, 0.979, and 0.969; 883, 0.917, 0.717, and 0.917	Machine learning classifiers based on radiomics, utilizing quantitative ADC and (CBF) maps, prove to be effective in distinguishing HGGs from LGGs.
Training cohort—101 patients; test cohort—50 glioma patients	Training cohort and test cohort	-	0.847 0.898	Imaging features extracted from multiplanar contrast-enhanced T1-weighted magnetic resonance imaging (CE-T1W MPR) are more effective than features from single planes in differentiating higher-grade gliomas (HGGs) and lower-grade gliomas (LGGs).
567 glioblastomas				The created model was equivalent to the clinical radiologists and performed better than the models based solely on radiomics or deep features (*p* < 0.001).
Training (n = 136) and test (n = 68) sets of glioma patients	Lower-grade glioma (LGG)	-	0.85	Classifiers based on radiomic features hold potential utility in predicting LGG grades.
30 patients (14 LGG and 16 HGG)				Automated tumor segmentation and extraction of radiomic features from combined [18F] FET PET and CE T1-WI MRI scans have demonstrated the ability to discriminate between lower-grade glioma (LGG) and higher-grade glioma (HGG).

Abbreviations: DTI: diffusion tensor imaging; DWI diffusion-weighted imaging; HGG: high-grade glioma; FA: fractional anisotropy; LR: logistic regression; LGG: low-grade glioma; MLP: multilayer perceptron; IS: image signature; RF: random forest; SVM: support vector machine.

### 3.4. Risk of Bias Assessment

The studies summarized in Table 1 and Table 2 exhibited an overall risk of bias. This elevated bias is likely associated with the retrospective nature of the studies, where the outcomes were already known. The evaluation method and the inclusion and exclusion criteria were explained prior to the enrolment of the participants in all the included studies. Overall, the studies demonstrated varying degrees of reporting and addressing key methodological aspects. However, none of the studies fully reported or addressed all assessed criteria, indicating potential risks of bias across the studies. The most reported and addressed aspects were related to discrimination statistics, with several studies providing information on discrimination metrics such as the sensitivity, specificity, and area under the receiver operating characteristic curve (AUC). However, other critical aspects such as phantom studies on all scanners, adjustment for multiple testing, and prospective study registration in a trial database were rarely reported or addressed. The lack of reporting and addressing key methodological aspects raises concerns about the robustness and generalizability of the study findings. The standardization and transparent reporting of methodologies, as well as rigorous validation of machine learning algorithms, are essential to mitigate bias and enhance the reliability of future studies in this field. None of the studies had cost-effectiveness analysis.

## 4. Discussion

In this systematic review, we synthesized evidence from 18 studies to assess the potential of radiomics in glioma grading and differential diagnosis. The included studies exhibited a wide range of characteristics in terms of study design, sample size, imaging modalities, and machine learning algorithms employed, collectively highlighting the versatility and promise of radiomics in glioma characterization.

The variety of methodologies used can complicate the generalizability of findings. Although deep learning models show considerable promise, their dependence on large datasets may restrict their applicability in smaller clinical settings. This diversity enhances the generalizability of findings across different settings and patient populations. Imaging modalities such as MRI, PET/CT, ASL, and DTI were employed, allowing for the comprehensive characterization of gliomas and enhancing diagnostic accuracy.

Furthermore, a variety of machine learning algorithms, including deep learning networks, support vector machines, random forests, and logistic regression, were utilized, reflecting the complexity of glioma classification and the need for robust predictive models to handle radiomic data intricacies.

Several studies demonstrated the effectiveness of radiomics-based machine learning models in glioma grading and differential diagnosis. For instance, Luo et al., 2020 [11], and Hashido et al., 2021 [19], utilized deep learning networks and radiomic features extracted from MRI maps to differentiate between glioma subtypes with high accuracy. Additionally, Zhu et al., 2023 [15], and Kumar et al., 2023 [20], investigated the performance of various machine learning algorithms in discriminating between HGGs and LGGs, showcasing the potential of radiomics for accurate classification.

Several studies, including those by He et al. [27], Hashido et al. [19], and Ning et al. [24], aimed to predict the molecular subtypes or mutation status of gliomas using radiomics and machine learning techniques. These studies demonstrated the feasibility of the noninvasive molecular characterization of gliomas, with accuracies ranging from 80% to 94%. Such approaches hold promise in guiding personalized treatment strategies and improving patient outcomes by enabling targeted therapies based on the molecular profile of the tumor.

Furthermore, Ding et al. [22], Bonte et al. [23], and Park et al. [25] investigated the combination of radiomics and deep learning techniques to enhance glioma grading accuracy. These studies showed that integrating deep learning features with radiomic features extracted from multiplanar reconstructed images or PET scans can improve the classification performance. However, some studies noted limitations in external validation, emphasizing the need for the further refinement and validation of these models in larger cohorts and diverse clinical settings.

Overall, the findings from this systematic review underscore the potential of radiomics and machine learning techniques in enhancing the accuracy and efficiency of glioma diagnosis, grading, and molecular characterization. However, the further validation and standardization of these approaches are necessary to ensure their clinical applicability and generalizability across different healthcare settings. Collaborative efforts involving multidisciplinary teams, large-scale datasets, and prospective studies are essential to advance the field and ultimately improve patient outcomes in glioma management.

## 5. Conclusions

In conclusion, the evidence synthesized from these studies highlights the potential of radiomics as a valuable tool for glioma grading and differential diagnosis. Radiomics-based machine learning models demonstrate high accuracy in distinguishing between glioma subtypes and grades, surpassing traditional imaging methods and inexperienced radiologists in some cases. Radiomics has the potential to completely transform the way gliomas are managed by directing treatment choices and enhancing patient outcomes with additional validation and standardization efforts. In order to make it easier for radiomics to be incorporated into regular clinical practice, future research should concentrate on prospective validation studies and the creation of uniform procedures.

## Data Availability

Not applicable.

## Supplementary Materials

The following supporting information can be downloaded at https://www.mdpi.com/article/10.3390/diagnostics14232741/s1. Text S1: Search strategy used in various databases; Table S1: Radiomics quality scores of the selected studies; Table S2: Radiomics quality score; Table S3: MRI parameters; Table S4: Description of the segmentation methods employed by previous studies.
