# Supplementary material for: Quality Assessment of MRI-Radiomics-Based Machine Learning Methods in Classification of Brain Tumors: Systematic Review
Source: Diagnostics (Basel). 2024 Dec 5;14(23):2741. doi: 10.3390/diagnostics14232741 (PMC11639919; doi:10.3390/diagnostics14232741)
Supplement: Supplementary file 1 [file diagnostics-14-02741-s001.zip › diagnostics-3261920-supplementary.pdf]

diagnostics

MDPI

Systematic Review

Quality Assessment of MRI-Radiomics-Based Machine Learning Methods in Classification of Brain Tumors: Systematic Review

Shailesh S. Nayak [1], Saikiran Pendem [1], Girish R. Menon [2], Niranjana Sampathila [3] and Prakashini Koteshwar [2,*]

Supplementary Materials: The following Search strategy used in various databases.

("brain"[MeSH Terms] OR "brain"[All Fields] OR "brains"[All Fields] OR "brain s"[All Fields]) AND ("brain tumour"[All Fields] OR "brain neoplasms"[MeSH Terms] OR ("brain"[All Fields] AND "neoplasms"[All Fields]) OR "brain neoplasms"[All Fields] OR ("brain"[All Fields] AND "tumor"[All Fields]) OR "brain tumor"[All Fields] OR ("glioma"[MeSH Terms] OR "glioma"[All Fields] OR "gliomas"[All Fields] OR "glioma s"[All Fields]) OR ("brain neoplasms"[MeSH Terms] OR ("brain"[All Fields] AND "neoplasms"[All Fields]) OR "brain neoplasms"[All Fields] OR ("intracranial"[All Fields] AND "tumor"[All Fields]) OR "intracranial tumor"[All Fields]) OR ("brain neoplasms"[MeSH Terms] OR ("brain"[All Fields] AND "neoplasms"[All Fields]) OR "brain neoplasms"[All Fields] OR ("brain"[All Fields] AND "neoplasm"[All Fields]) OR "brain neoplasm"[All Fields]))

"machine learning"[MeSH Terms] OR ("machine"[All Fields] AND "learning"[All Fields]) OR "machine learning"[All Fields] OR ("artificial intelligence"[MeSH Terms] OR ("artificial"[All Fields] AND "intelligence"[All Fields]) OR "artificial intelligence"[All Fields])

(("magnetic resonance spectroscopy"[MeSH Terms] OR ("magnetic"[All Fields] AND "resonance"[All Fields] AND "spectroscopy"[All Fields]) OR "magnetic resonance spectroscopy"[All Fields] OR ("magnetic"[All Fields] AND "resonance"[All Fields]) OR "magnetic resonance"[All Fields]) AND ("radiomic"[All Fields] OR "radiomics"[All Fields])) OR ("radiomic"[All Fields] OR "radiomics"[All Fields]) OR ("Machine-Learning-Based"[All Fields] AND ("radiomic"[All Fields] OR "radiomics"[All Fields]))

"classification"[MeSH Terms] OR "classification"[All Fields] OR "classifications"[All Fields] OR "classification"[MeSH Subheading] OR "classification s"[All Fields] OR "classificator"[All Fields] OR "classificators"[All Fields] OR ("diagnosable"[All Fields] OR "diagnosi"[All Fields] OR "diagnosis"[MeSH Terms] OR "diagnosis"[All Fields] OR "diagnose"[All Fields] OR "diagnosed"[All Fields] OR "diagnoses"[All Fields] OR "diagnosing"[All Fields] OR "diagnosis"[MeSH Subheading]) OR ("diagnosis, differential"[MeSH Terms] OR ("diagnosis"[All Fields] AND "differential"[All Fields]) OR "differential diagnosis"[All Fields] OR ("differential"[All Fields] AND "diagnosis"[All Fields]))

("brain"[MeSH Terms] OR "brain"[All Fields] OR "brains"[All Fields] OR "brain s"[All Fields]) AND ("brain tumour"[All Fields] OR "brain neoplasms"[MeSH Terms] OR ("brain"[All Fields] AND "neoplasms"[All Fields]) OR "brain neoplasms"[All Fields] OR ("brain"[All Fields] AND "tumor"[All Fields]) OR "brain tumor"[All Fields] OR ("glioma"[MeSH Terms] OR "glioma"[All Fields] OR "gliomas"[All Fields] OR "glioma s"[All Fields]) OR ("brain neoplasms"[MeSH Terms] OR ("brain"[All Fields] AND "neoplasms"[All Fields]) OR "brain neoplasms"[All Fields] OR ("intracranial"[All Fields]

AND "tumor"[All Fields]) OR "intracranial tumor"[All Fields]) OR ("brain neoplasms"[MeSH Terms] OR ("brain"[All Fields] AND "neoplasms"[All Fields]) OR "brain neoplasms"[All Fields] OR ("brain"[All Fields] AND "neoplasm"[All Fields]) OR "brain neoplasm"[All Fields])) AND ("machine learning"[MeSH Terms] OR ("machine"[All Fields] AND "learning"[All Fields]) OR "machine learning"[All Fields] OR ("artificial intelligence"[MeSH Terms] OR ("artificial"[All Fields] AND "intelligence"[All Fields]) OR "artificial intelligence"[All Fields])) AND ((("magnetic resonance spectroscopy"[MeSH Terms] OR ("magnetic"[All Fields] AND "resonance"[All Fields] AND "spectroscopy"[All Fields]) OR "magnetic resonance spectroscopy"[All Fields] OR ("magnetic"[All Fields] AND "resonance"[All Fields]) OR "magnetic resonance"[All Fields]) AND ("radiomic"[All Fields] OR "radiomics"[All Fields])) OR ("radiomic"[All Fields] OR "radiomics"[All Fields]) OR ("Machine-Learning-Based"[All Fields] AND ("radiomic"[All Fields] OR "radiomics"[All Fields]))) AND ("classification"[MeSH Terms] OR "classification"[All Fields] OR "classifications"[All Fields] OR "classification"[MeSH Subheading] OR "classification s"[All Fields] OR "classificator"[All Fields] OR "classificators"[All Fields] OR ("diagnosable"[All Fields] OR "diagnosi"[All Fields] OR "diagnosis"[MeSH Terms] OR "diagnosis"[All Fields] OR "diagnose"[All Fields] OR "diagnosed"[All Fields] OR "diagnoses"[All Fields] OR "diagnosing"[All Fields] OR "diagnosis"[MeSH Subheading]) OR ("diagnosis, differential"[MeSH Terms] OR ("diagnosis"[All Fields] AND "differential"[All Fields]) OR "differential diagnosis"[All Fields] OR ("differential"[All Fields] AND "diagnosis"[All Fields])))

Table S1: Radiomics quality scores of the selected studies.

RQS item		Score
Image protocol quality	Well-documented image protocols (for example, contrast, slice thickness, energy, etc.) and/or usage of public image protocols allow reproducibility/replicability	+ 1 (if protocols are well-documented)<br>+ 1 (if public protocol is used)
Multiple segmentations	Segmentation by different physicians/algorithms/software, perturbing segmentations by (random) noise, segmentation at different breathing cycles. Analyse feature robustness to segmentation variabilities	+ 1
Phantom study on all scanners	Detect inter-scanner differences and vendor-dependent features. Analyse feature robustness to these sources of variability	+ 1
Imaging at multiple time points	Collect images of individuals at additional time points. Analyse feature robustness to temporal variabilities (for example, organ movement, organ expansion/ shrinkage)	+ 1
Feature reduction or adjustment for multiple testing	Decreases the risk of overfitting. Overfitting is inevitable if the number of features exceeds the number of samples. Consider feature robustness when selecting features	- 3 (if neither measure is implemented) + 3 (if either measure is implemented)
Validation	The validation is performed without retraining and without adaptation of the cut-off value, provides crucial information with regards to credible clinical performance	- 5 (if validation is missing)<br>+ 2 (if validation is based on a dataset from the same institute)<br>+ 3 (if validation is based on a dataset from another institute)<br>+ 4 (if validation is based on two datasets from two distinct institutes)<br>+ 4 (if the study validates a previously published signature)

		+ 5 (if validation is based on three or more datasets from distinct institutes)
Multivariable analysis with non-radiomics features	(for example, EGFR mutation) - is expected to provide a more holistic model. Permits correlating/inferencing between radiomics and non-radiomic features	+1
Detect and discuss biological correlates	Demonstration of phenotypic differences (possibly associated with underlying gene–protein expression patterns) deepens understanding of radiomics and biology	+1
Comparison to 'gold standard'	Assess the extent to which the model agrees with/is superior to the current 'gold standard' method (for example, TNM-staging for survival prediction). This comparison shows the added value of radiomics	+2
Potential clinical utility	Report on the current and potential application of the model in a clinical setting (for example, decision curve analysis).	+2
Cut-off analyses	Determine risk groups by either the median, a previously published cut-off or report a continuous risk variable. Reduces the risk of reporting overly optimistic results	+1
Discrimination statistics	Report discrimination statistics (for example, C statistic, ROC curve, AUC) and their statistical significance (for example, p values, confidence intervals). One can also apply resampling method (for example, bootstrapping, cross-validation)	+ 1 (if a discrimination statistic and its statistical significance are reported) + 1 (if a resampling method technique is also applied)
Calibration statistics	Report calibration statistics (for example, Calibration-in the-large/slope, calibration plots) and their statistical significance (for example, P values, confidence intervals). One can also apply resampling method (for example, bootstrapping, cross-validation)	+ 1 (if a calibration statistic and its statistical significance are reported) + 1 (if a resampling method technique is also applied)
Prospective study registered in a trial database.	Provides the highest level of evidence supporting the clinical validity and usefulness of the radiomics biomarker	+ 7 (for prospective validation of a radiomics signature in an appropriate trial)
Cost-effectiveness analysis	Report on the cost-effectiveness of the clinical application (for example, QALYs generated)	+1
Open science and data	Make code and data publicly available. Open science facilitates knowledge transfer and reproducibility of the study	+ 1 (if scans are open source) + 1 (if region of interest segmentations are open source) + 1 (if code is open source) + 1 (if radiomics features are calculated on a set of representative ROIs and the calculated features and representative ROIs are open source)

Table S2: Radiomics quality score

**Author**	**Image protocol quality**	**Multiple segmentations**	**Phantom study on all scanner**	**Imaging at multiple time points**	**Feature reduction or adjustment for multiple testing**	**Multivariable analysis with non radiomics features**	**Detect and discuss biological correlation**	**Cut - off analyse**	**Discrimination statistics**	**Calibration statistics**	**Prospective study registered in a trial database**	**Validation**	**Comparison to gold standard**	**Potential clinical utility**	**Cost-effectiveness ss analysis**	**Open science and data**	**Total**
**Luo.et.al., 2020 [11]**	1	1	0	0	1	0	0	1	1	0	1	0	1	1	0	0	8
**Takahiro Nakamoto . et.al., 2019 [12]**	1	0	0	1	1	1	0	0	1	0	0	1	1	1	0	0	8
**Gutta.et.al .,2021 [13]**	1	0	0	0	1	0	0	0	1	0	0	0	0	1	0	0	4
**Russo.et.al .,2021 [26]**	2	2	1	1	1	1	1	1	1	1	0	1	2	1	0	1	18
**Zhang.et.al., 2020 [14]**	1	1	0	0	3	1	0	0	2	1	0	3	2	2	0	0	16 (44%)
**Zhu.et.al., 2023 [15]**	1	1	0	0	0	2	2	0	2	0	0	2	2	2	0	0	14
**Guo.et.al., 2023 [17]**	1	0	0	0	1	0	0	1	0	0	0	1	0	1	0	0	6
**Kumar.et.al.,2023 [20]**	2	0	0	0	2	2	2	2	2	2	0	2	2	2	0	1	21
**He.et.al.,2022 [27]**	2	0	0	0	1	0	0	0	1	0	0	0	0	1	0	0	5
**J.et.al.,2022 [21]**	2	0	0	0	1	0	0	0	1	0	0	1	0	1	0	0	6
**Guo.et.al., 2021 [28]**	2	0	0	0	1	1	0	0	1	0	0	1	0	1	0	0	7
**Zhang.et.al.,2021 [18]**	1	2	0	0	2	2	0	0	2	0	0	2	2	2	0	2	17
**Hashido.et.al.,2021 [19]**	2	2	0	0	2	2	0	0	2	0	0	2	2	2	0	2	18
**Ding.et.al.,2022 [22]**	2	2	0	0	2	2	0	0	2	0	0	2	2	2	0	2	18

**Ning.et.al. ,2021 [24]**	2	2	0	0	2	2	0	0	2	0	0	2	2	2	0	2	18
**Park.et.al., 2019 [25]**	2	2	0	0	2	2	0	0	2	0	0	2	2	2	0	2	18
**Bonte.et.al .,2018 [23]**	2	2	0	0	2	2	0	0	2	0	0	2	2	2	0	2	18

Table S3: MRI Imaging Parameters

**Author and Year**	**MR Imaging Parameters**						
	**Contrast**	**Slice thickness and Spacing (mm)**	**Matrix size, Pixel size (mm^2)**	**TR (ms)**	**TE (ms)**	**FOV (mm)**	**Flip angle (degree)**
Luo.et.al., 2020 [11]	CE T1 and T2 FLAIR series of MRI images	Imaging parameters unavailable.					
Takahiro Nakamoto. et.al., 2019 [12]	CE-T1WI	5.0 and 6.0	256 × 256/ 0.9 × 0.9	640	8–12	-	-
	T2WI	3.0 and 3.0	512 × 512/ 0.5 × 0.5	4500	80–90	-	-
Gutta.et.al.,2021 [13]	T1-WI	5 &7	-	700	10	-	90
	CE-T1WI	5 &7	-	500	19	-	90
	T2-WI	5 &7	-	5000	100	-	90
	T2 FLAIR	5 &7	-	8802	158	-	90
Giorgio Russo.et.al.,2021 [26]	11[C]-MET PET/CT	3.27& 1.17	512×512	-	-	-	-
Zhang.et.al., 2020 [14]	T1-WI IR	-	-	-	-	-	-
	T2-WI	-	-	-	-	-	-
	FLAIR	-	-	-	-	-	-
	T1-CEWI	-	-	-	-	-	-
	DTI SS-EPI (b = 1000 and 0 s/mm2)	5.0 & 0	128 × 128	8000	88	240× 240	-
Zhu.et.al.,2023 [15]	Siemens 1.5T MRI: T1WI-CE	5	256×256	339	3.54	-	-
	General Electricals 3T:	5	256×256	1750	25	-	-
	T1WI-CE	4		4376	11	-	111
	3D-ASL						
Kumar.et.al.,2023 [20]	GE Signa 3T: T2-WI	5:1.5	320×224	4080	90	24	-
		5:1	448×304	6000	107	23	-

	Philips Ingenia 1.5T: T2-WI						
He.et.al.,2022 [27]	T1-WI	Study included public dataset; the imaging parameters are unavailable.					
	T2-WI						
	FLAIR						
	CE T1- WI						
J. et.al.,2022 [21]	CE-T1WI	4	128×128-	5000	90	-	90
	T2WI FLAIR	6	256×256	3000	105	-	90
	DWI sequence	-	192×192	-	-	-	-
Guo.et.al.,2021 [28]	CE-T1WI	6 & 1.2-1.8	320×320	250	2-2.50	240×240	70
	T2 WI	6-6.5& 1.8	320×320	3500	113	240×240	90
	FLAIR	6 & 1.2-1.8	256×162	8000	94-97	240×240	130
	DWI (b-value-0, 1000)	6 & 1.2-1.8	128×128	3000	54-82	240×240	90-180
Zhang.et.al.,2021 [18]	T1-WI	Study included public dataset; the imaging parameters are unavailable.					
	T2-WI						
	CE T1-WI						
	FLAIR						
Hashido.et.al.,2021 [19]	T1 WI FLAIR	5 & 1	352×256	2600	24	220	111
	T2 WI FLAIR	5 & 1	320×224	8800	140	220	111
	T2 WI	5 & 1	512×320	5800	96	220	111
	T2*WI	5 & 1	480×224	770	18	220	20
	CE T1 WI	0.9	512×320	8.8	2.8	240	15
Ding.et.al.,2022 [22]	T1 FSE	5	512×512	2.5	2.3	-	-
	CE T1-WI	3.2	512×512	3.8	1.7	-	-
Ning.et.al.,2021 [24]	T1-WI	6	512×512	214	4.6	82%	80
	T2-WI FLAIR	5	256×256	600	17	75%	90
Park.et.al.,2019 [25]	T1-WI	5	256 x 256	1800	10–15	240	-
	T2-WI	5	256 x 256	2800	90	240	-
	FLAIR	5	256 x 256	9500	120	240	-
	3D CE T1-WI	1	192 x 192	7	3.5	240	-

Table S4: Description of the segmentation methods employed by previous studies.

Author and Year	Validation tool	Segmentation method employed	Data sets used for validation
Luo.et.al., 2020 [11]	DL network (3D U-net) and IS	Tumors in the training dataset were manually delineated by two experienced neurosurgeons, with these segmentations serving as the ground truth for training the 3D U-Net model.	The dataset for deep learning training, comprising 188 cases, was randomly divided into a training set of 125 cases and a validation set of 63 cases, maintaining a 2:1 ratio.

Takahiro Nakamoto. et.al., 2019 [12]	LR, SVM43, SNN44, RF45, and NB4	Tumor segmentations were generated using a computerized framework and subsequently corrected by a neuroradiologist.	An independent validation was conducted to assess the versatility of radiomic analysis in predicting malignant glioma grades using a limited number of structural MRI sequences across both the primary and validation datasets.
Gutta.et.al,2021 [13]	SVM, RF, and gradient boosting trained with radiomic feature	A fully automated brain tumor segmentation tool was employed to identify lesions, and all segmentations were visually reviewed by a board-certified neuroradiologist with nine years of experience.	Convolutional network was used to classify glioma grades based on pre- and post-CE MR images, with performance compared against three established machine learning methods.
Giorgio Russo.et.al,2021 [26]	Neural networks, RF, SVM, and generalized linear models	LIFEx software was utilized to perform the lesion delineation step within the radiomics workflow.	Each dataset was divided into subsets, with one subset designated as the validation set and the remaining subsets combined to form the training set.
Zhang.et.al, 2020 [14]	SVM classifier with a linear kernel	Tumor regions were manually segmented slice by slice using ITK-SNAP software by an experienced radiologist with 14 years of experience and reviewed by another senior radiologist.	Leave-one-out cross-validation was utilized to evaluate the performance of the classification models, aiming to assess the model's ability to predict new data and provide insight into how it would generalize to an independent dataset.
Zhu.et.al., 2023 [15]	MLP, SVM, RF and logistic regression	The tumor regions were manually delineated by one to four experienced radiologists.	The data were divided into training and verification groups in an 8:2 ratio.
Kumar.et.al.,2023 [20]	RF Classifier RFC, SVM, GBC, NBC, and ABC.	Tumor segmentation and region of interest (ROI) delineation were performed using the semiautomated segmentation tool and segmentation verified by a neuro-oncologist and a neuroradiologist, both with 10 years of experience.	Model validation was performed using 10-fold internal cross-validation, where the training data were split into ten subsets, with one subset reserved for testing and the model trained on the remaining nine subsets.
He.et.al., 2022 [27]	SVM, AE, RF, LDA, LR, LR via lasso (LR-Lasso), & DT	A public retrospective dataset was used, consisting of 108 patients, with manually corrected segmentations employed for feature extraction.	The random classification method divided the dataset into a training set and a test set with an 8:2 ratio, consisting of 80 training samples and 19 testing samples.
J. et.al., 2022 [21]	Monoexponential, IVIM, DKI, FROC, CTRW, stretched exponential using MATLAB	Two radiologists placed regions of interest (ROIs) on the solid portions of tumors in the DWI images, carefully avoiding areas of necrosis, edema, and hemorrhage.	Prediction models were initially trained using five-fold cross-validation. The final prediction models for the three classification methods were then selected based on the highest AUC in the internal test set.
Guo.et.al., 2021 [28]	LASSO	The segmentation of the VOIs was performed independently by a senior neuroradiologist, with inter-reader consistency verified by segmenting the VOIs of 30 randomly selected	The patients were divided into a training cohort and a validation cohort in a 7:3 ratio.

		cases also by another senior neuro-radiologist.	
Zhang.et.al.,2021 [18]	RF classification algorithm	MRI image segmentation was performed using a computer-aided method and subsequently corrected manually, with the lesion segmented into edema, tumor contrast-enhanced, and non-contrast-enhanced areas.	This study included 102 patients with GBM and 65 patients with LGG from the open-source TCIA database, with the patients divided into two groups: the training set and the external test set.
Hashido.et. al., 2021 [19]	LASSO-LR, RF, SVM with the radial basis function kernel (SVM-RBF), and SVM with the linear kernel (SVM-L)	The VOI was semi-automatically segmented using a supervised random forests (RFs) machine learning algorithm implemented in ITK-SNAP.	Patient data were randomly divided into 70% for the training set and 30% for the independent test set, with four types of machine learning classifiers trained to predict glioma grade (LGG or HGG) using radiomic features
Ding.et.al., 2022 [22]	SVM, LR and RF	The gross tumor volumes (GTVs) were manually delineated on the axial MPR images by an oncologist using 3D Slicer software, after which the contoured GTVs were reviewed slice-by-slice by another oncologist with 5 years of experience.	A total of 131 patients were selected for the training and test1 cohorts, while 20 patients from the open-source Cancer Imaging Archive were chosen for the test2 cohort, with test1 and test2 subsequently merged to form the final test set.
Ning.et.al., 2021 [24]	A kernel fusion based SVM classifier was used to integrate these multi-modal features for grading gliomas.	The VOIs were manually delineated on T2 FLAIR images using ITK-SNAP 3.6 by a radiologist with 10 years of experience.	A total of 567 patients were enrolled and divided into training (n=186), validation (n=47), and testing (n=334) cohorts.
Park.et.al., 2019 [25]	Elastic net, RF, GBM, and LDA algorithms,	Data from institutional and open-source databases TCGA/TCIA were collected, and tumor lesions on the images were segmented by two neuroradiologists with 10 and 7 years of experience, respectively.	A total of 204 patients were enrolled in the institutional cohort, which was semi-randomly allocated into training (n = 136) and test (n = 68) sets, while data from open-source databases TCGA/TCIA were used for validation.
